# Linear Accelerator (LINAC) Radiosurgical Management of Brain Arteriovenous Malformations: An Experience From a Tertiary Care Center

**DOI:** 10.7759/cureus.76232

**Published:** 2024-12-22

**Authors:** Amith Mohan, Sarbesh Tiwari, Puneet Pareek, Antonio Fernandes, Sanjay Santhyavu, Sri Harsha Kombathula, Mukul Choubisa, Sanjib Gayen, Mohammed Irfad, Akanksha Solanki

**Affiliations:** 1 Radiation Oncology, All India Institute of Medical Sciences, Jodhpur, Jodhpur, IND; 2 Radiodiagnosis, All India Institute of Medical Sciences, Jodhpur, Jodhpur, IND; 3 Medical Physics, All India Institute of Medical Sciences, Jodhpur, Jodhpur, IND

**Keywords:** avm radiosurgery, brain avm, frameless radiosurgery, linac radiosurgery, radiosurgery outcome

## Abstract

Introduction: Brain arteriovenous malformations (AVM) are complex vascular pathologies with a significant risk of hemorrhage. Stereotactic radiosurgery (SRS) is an effective treatment modality for AVM, initially popularized on the Gamma Knife (Elekta AB, Stockholm, Sweden) platform, and now benefits from the modern advances in linear accelerator (LINAC)-based platforms. This study evaluates the outcomes of LINAC-based SRS/hypofractionated stereotactic radiotherapy (hFSRT) for cerebral AVMs.

Materials and methods: Between December 2018 and April 2024, 15 patients with cerebral AVMs underwent SRS/hFSRT at a tertiary government hospital. Patient selection was based on AVM size, location, surgical unsuitability, and patient preference. All patients underwent MRI and cerebral angiography for nidus delineation. SRS was planned using Monaco TPS (treatment planning system) (Elekta AB, Stockholm, Sweden) with VMAT (volumetric modulated arc therapy) technique, delivering a median dose of 20 Gy in single fractions for small AVMs and 28 Gy in four fractions for large AVMs. Patients were followed up with annual MRI and angiography to assess obliteration.

Results: The cohort had a median age of 22 years, with a median nidus volume of 3.76 cc. The crude obliteration rate was 60%, confirmed by MRI/angiography. Actuarial obliteration rates at two, three, and five years were 65.71%, 73.57%, and 77.14%, respectively. Smaller AVMs (<3 cc) and those with a modified AVM radiosurgery score <1.5 had nearly 100% obliteration rates. Large AVMs (>10 cc) treated with hypofractionated SRT showed partial responses only. Significant predictors of obliteration included prescription dose, AVM volume, and modified AVM radiosurgery score.

Conclusion: LINAC-based SRS demonstrates comparable efficacy to other modalities for treating cerebral AVMs, with obliteration rates influenced by dose, AVM volume, and pre-treatment radiosurgery score. Larger AVMs pose a greater challenge, suggesting a need for adjunctive treatments or higher fractionated doses to improve outcomes.

## Introduction

Arteriovenous malformations (AVMs) are vascular malformations in which arterial blood is directly shunted into the venous system under high pressure via a vascular malformation or nidus by-passing normal capillary system. The estimated prevalence of symptomatic AVM is 0.94 per 100000 person-years [1]. They carry an innate risk of intracranial hemorrhage associated with neurocognitive deficits, morbidity, and mortality. This risk of hemorrhage is significant, estimated at 2-4% per year in an unruptured AVM, and can reach up to 6% the year after any hemorrhagic episodes [2].

Managing AVMs is challenging for physicians, who must consider the natural history of hemorrhage and associated morbidity and mortality, alongside potential treatment toxicity. Several management options are available, including observation, embolization, and excision. Stereotactic radiosurgery (SRS) was introduced as a treatment option in the 1970s and has since been developed into a minimally invasive technique that aims to obliterate the AVM nidus, thereby eliminating the risk of future hemorrhage and having an acceptable side effect profile [3].

Earlier literature is predominantly based on Gamma Knife (Elekta AB, Stockholm, Sweden) SRS, which was also, the only reliable option in that period. The modern modifications to the linear accelerator (LINAC) platform like the micro multileaf collimator (mMLC), onboard imager (CBCT, i.e., cone beam computed tomography), non-co-planar beam arrangements, reliable frameless immobilization, and advanced planning algorithms have transformed it into a reliable and effective radiosurgery platform [4]. The modern Gamma Knife (Elekta AB) systems like Icon (Elekta AB, Stockholm, Sweden) have adaptations like inverse treatment planning (lightning planning system), onboard CBCT imaging and frameless immobilization, and fractionated treatment for larger volumes, which were features already in use on the LINAC platforms [5,6]. However, the use of LINAC-based SRS for cerebral AVMs is not that common among radiation oncologists and neurosurgeons, despite its wide availability. In this study, we aimed to evaluate the outcomes of LINAC-based SRS for cerebral AVMs and review the relevant literature.

## Materials and methods

This is a prospective interventional study evaluating the treatment outcome of 15 arteriovenous malformation (AVM) patients treated between 2018 and 2024, using LINAC-based SRS/hypofractionated stereotactic radiotherapy (hFSRT).

Patient population and selection

Between December 2018 and April 2024, a total of 15 patients with one AVM nidus in each, i.e. 15 AVM targets underwent SRS at the Department of Radiation Oncology, All India Institute of Medical Sciences, Jodhpur (AIIMS, Jodhpur) (tertiary care government health center). The Institutional Ethics Committee, AIIMS, Jodhpur issued approval AIIMS/IEC/2018/782, dated December 22, 2018. All patients were evaluated and chosen for treatment at a joint neuroradiology and radiation oncology outpatient clinic. AVMs were selected for radiosurgery based on their size, intracerebral location, and eloquence, proximity to critical OAR (organs at risk), and unsuitability for surgery due to the risk of neurological deficits or patient preference.

*Inclusion Criteria* 

Confirmed diagnosis: Patients with brain AVMs confirmed by MRI and/or digital subtraction angiography (DSA).

Size and location criteria: AVMs with a nidus size ≤10 cc was selected for SRS and those with >10cc were selected for hFSRT. AVMs located within 3 mm of optic chiasm were also considered for hFSRT.

Clinical suitability: AVMs deemed unsuitable for surgical resection due to high surgical risk or patient preference (patients who opted for radiosurgery after being informed of alternative treatment options).

Informed consent: Patients (or guardians, if applicable) who provided written informed consent for treatment and follow-up.

Follow-up commitment: Patients willing and able to comply with follow-up protocols, including periodic MRI or DSA.

*Exclusion Criteria* 

Medical contraindications: Patients with severe neurological complications, making them unsuitable for immobilization.

Unacceptable AVM characteristics: AVMs with diffuse or poorly delineated nidus on imaging, making precise targeting impossible.

Pregnancy or lactation: Pregnant or lactating individuals at the time of treatment.

Refusal of follow-up or consent: Patients unwilling to undergo required imaging follow-ups or provide consent for participation.

All patients underwent diagnostic magnetic resonance imaging (MRI) and cerebral angiography before treatment. All patients underwent thorough neurological examination and audiometric and visual perimetry evaluations prior to SRS/hFSRT. In certain cases, patients underwent embolization prior to radiation therapy, as deemed necessary by the interventional radiology department.

Imaging and segmentation

Computed tomography (CT) simulation was performed using a General Electric (GE) Optima 580RT machine (GE HealthCare, Boston, MA) and scans were obtained with a slice thickness of 1 mm. All patients were immobilized using a double-shell positioning system (DSPS) thermoplastic mask and frame (MacroMedics, Moordrects, The Netherlands), and three orthogonal fiducials were placed. On the same day, MRI was also done at 1 mm slice thickness using 3 Tesla (3T) GE MRI (GE HealthCare, Boston, MA), and the MRI sequences used were T1BRAVO pre- and post-contrast, time-of-flight (TOF), and T2 cube. Following this the MR and CT images were imported, co-registered and fused in Monaco TPS (Treatment Planning System) (Elekta AB, Stockholm, Sweden), and then the image fusion accuracy was confirmed manually by the neuroradiologist and radiation oncologist. The nidus was delineated on MR sequences such as TOF and post-contrast T1 BRAVO, and the DSA (digital subtraction angiography) images were evaluated alongside for delineation of AVM but were not co-registered.

Classification of AVM

The AVM nidus was classified as per the SM (Spetzler-Martin system) grade based on the DSA imaging characteristics like size (diameter in centimeters), location (eloquence) and drainage pattern (deep vs superficial) [7]. Similarly, after generating the final nidus volume in TPS, the obliteration probability and outcome scores were calculated as per the modified radiosurgery-based AVM grading scale,

(AVM score = (0.1)(volume, cc) + (0.02)(age, years) + (0.5)(location; frontal/temporal/parietal/occipital/intraventricular/corpus callosum/cerebellar = 0, basal ganglia/thalamus/brainstem = 1) [8].

Also, the AVMs were classified based on volumes into small (<3 cc), medium (3.1 cc - 10 cc) and large (>10 cc) [9].

Treatment planning

Treatment planning was performed using Monaco TPS (Elekta AB, Sweden) using the VMAT (volumetric modulated arc therapy) technique with noncoplanar multi-arc arrangements, with a 6MV beam with or without the flattening filter-free (FFF) technique. The final target volume was defined as the AVM nidus, the planning target volume (PTV) was kept the same as the AVM nidus, and no further margin was given. Plan evaluation was performed according to the standard criteria, parameters such as conformality index, homogeneity index, and gradient index were calculated, and the most suitable plan was approved. In the plan optimization, the dose to the whole brain minus PTV was given significant priority. Subsequently, thorough plan-specific QA (quality assurance) was performed. Finally, the treatment (SRS/FSRT) was delivered using LINAC (Versa HD Elekta) with a micro multileaf collimator (Agility, Elekta AB, Stockholm, Sweden) with a collimator width of 5 mm at isocenter. Patient positioning and verification were performed and confirmed using onboard CBCT (cone beam computed tomography) and room lasers. CBCT-based imaging verification was repeated for each fraction in hFSRT cases.

The selection of radiation dose and prescription isodose lines was based on various factors like the volume of the nidus, its location, and the V12 Gy (whole brain - nidus) achieved on each prescription which was accepted at <10 cc. No additional margin was given to the nidus target volume. Generally, the optimal dose is chosen to minimize the risk of radionecrosis or neurological injury, which typically ranges from 16 to 24 Gy for patients who had not been previously treated with radiation and had small lesions in non-eloquent areas. The dose selection was based on the studies by Flickinger et al. [10]. Patients with small AVM (less than 10 cc) received single-fraction SRS, and large AVM (>10.1 cc) were treated using hFSRT.

Patient follow-up

All the patients were prospectively monitored at the Radiation Oncology Department, annually, or more frequently if clinically necessary. Neurological symptoms, clinical signs, and treatment complications were documented during each visit. Six-monthly MRI scans were performed in the first year, followed by annual MRI scans to assess the response. The disappearance of flow voids within the AVM area was initially observed on follow-up MRI and was later confirmed through digital subtraction angiography. The absence of a nidus and early filling draining veins were indicative of AVM obliteration. AVMs were defined as obliterated if confirmed by angiography. Patients without AVM obliteration were generally followed up annually. If AVM obliteration was not achieved after four years of follow-up, the nidus was deemed patent.

Statistical method

After completing the descriptive statistics, variables such as dose, volume, baseline AVM radiosurgery score, and prior embolization status were analysed after grouping them into categorical variables using the chi-square test to evaluate their association with final obliteration achieved. Kaplan-Meier model was used to evaluate the time to obliteration. The small sample size made it difficult to evaluate the correlation and regression of the variables with the outcome.

## Results

The study comprised 15 individuals, including seven women and eight men, with a median age of 22 years (range, 10-60 years) at the time of intervention. The initial presentation in 93% (14/15) of the patients was cerebral hemorrhage, which presented symptomatically as headache in 93.3% (14/15). Approximately 40% (6/15) of the participants had neurological deficits related to arteriovenous malformations (AVM) (Table 1). Forty percent (6/15) of the patients had undergone prior embolization for the AVM.

**Table 1:** Patient characteristics. AVM: arteriovenous malformations.

Description		N (%)
Gender	Male	8 (53%)
	Female	7 (46%)
Bleed at presentation		14 (93%)
Presenting symptoms	Headache	14 (93%)
	Seizure	6 (40%)
	Vomiting	6 (40%)
	Loss of consciousness/altered sensorium	8 (53%)
	Motor weakness	6 (40%)
	Visual symptoms	2 (13%)
Neurological signs before treatment	Motor signs	5 (33%)
	Visual signs	3 (20%)
	Cranial nerve involvement	1 (6%)
	Cerebellar signs	1 (6%)
Location	Cortical	6 (40%)
	Periventricular/pericallosal	6 (40%)
	Basal ganglia	2 (13%)
	Cerebellum	1 (6%)
Intranidal aneurysms		4 (26%)
Modified AVM radiosurgery score	<=1	9 (60%)
	1.01-1.59	1 (6%)
	1.6-2	2 (13%)
	>2	3 (20%)

Of the AVMs, 93.3% (14 out of 15) were located in the supratentorial region, while only one AVM was found in the cerebellum. Among the supratentorial AVM, 57% (8/14) were located at the periventricular or pericallosal locations. The median volume of the nidus, as seen on MRI, was 3.76 cc (0.48 cc to 28 cc). The volume was determined after contouring using the treatment planning system; thus, it was determined that 40% were small (<3 cm3), 40% were medium (3.1-10 cm3), and 20% were large (>10.1 cm3). The median Spetzler-Martin score was 3(1-4) and the median modified AVM radiosurgery score was 0.957 (0.48 - 3.68).

Among the 15 patients, those with small or medium volume (12) were treated with SRS, and the three large AVMs were treated with hFSRT. For SRS, the median prescription dose was 20 Gy (range, 18-22 Gy), and the median marginal dose was 19.3Gy (16.7Gy - 22.3Gy), and all three large AVMs were treated with hFSRT, with a prescription of 28 Gy in four divided fractions over four consecutive days. The analysis of the prescription isodose lines across the treated cases revealed a range of values, with percentages varying from 58.55% to 80.62%.

Obliteration rate

**Figure 1:**
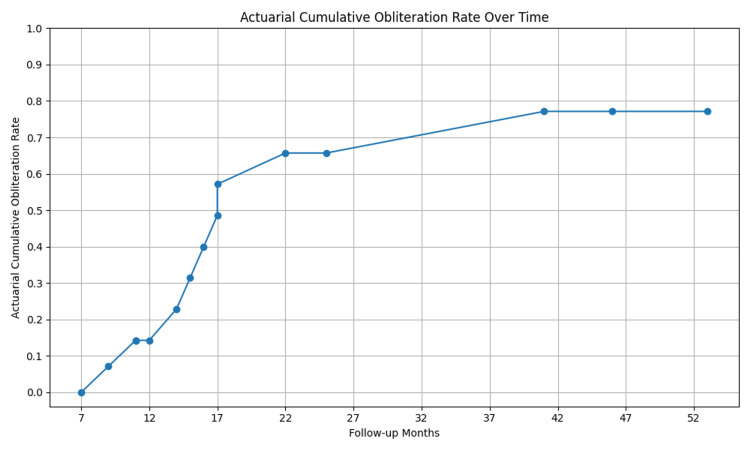
: Actuarial cumulative obliteration rate of AVM treated with SRS/hFSRT. AVM: arteriovenous malformations, SRS: stereotactic radiosurgery, hFSRT: hypofractionated stereotactic radiotherapy.

The crude obliteration rate (DSA confirmed) achieved using SRS/hFSRT for AVMs was 46% (7/15). When assessed with MRI or angiography (including one patient with a patent draining vein), the rate was 60% (9/15), and when excluding the patient who defaulted, the rate was 64% (9/14). The median follow-up period after radiation was 15 months (range: seven months to 53 months). The two-year, three-year, and five-year actuarial obliteration rates (MRI and/or angiography) were 65.71%, 73.57%, and 77.14%, respectively (Figure 1). For patients who completed adequate follow-up of at least four years, AVM volume of < 3 cc (small) and 3.1-10 cc (medium) led to an obliteration rate of 100% in both groups. In addition, for patients who completed adequate follow-up for at least four years with a modified AVM SRS score of < 1.5, an obliteration of 90% (MRI and/or angiography) was observed (Table 2). The median time to obliteration among the obliterated patients was 15 months (12-36 months). In addition, all three large AVMs that were treated with hFSRT only had a partial response and were patent at the end of three years.

**Table 2:** Obliteration rates relative to AVM volume and modified AVM SRS score for patients after adequate follow up (three years). AVM: arteriovenous malformations, SRS: stereotactic radiosurgery.

Parameter	Measure	Obliteration rate
AVM volume	<3 cc	100% (4/4)
	3-10 cc	100% (5/5)
	>10 cc	0% (0/3)
Modified AVM radiosurgery score	<1	88.88% (8/9)
	1-<1.5	100% (1/1)
	<1.5	90% (9/10)
	1.5<2	0(0/2)
	<2	75% (9/12)
	>=2	0(0/3)

The study assessed the association between prescription dose (EQD2, equivalent dose at 2Gy per fraction) (the doses were converted to EQD2, for the ease of including hypofractionated dose into the calculation), modified AVM radiosurgery score, prior embolization status, and volume with the final obliteration status for the AVMs (Table 3). The chi-square test for various volume groups (small, medium, and large) yielded a statistic of 5.972 with a corresponding p-value of 0.0505, suggesting that there might be a meaningful relationship between volume groups and obliteration status outcome. In addition, more complete obliteration events among small and medium samples were suggestive of better outcomes in this group.

**Table 3:** Association between clinical factors and AVM obliteration after primary SRS. EQD2: equivalent dose at 2 Gy per fraction, AVM: arteriovenous malformations, SRS: stereotactic radiosurgery.

Predictors	Chi-square statistics	p-value
EQD2	5.128	0.024
Modified AVM radiosurgery score	11.30	0.0102
Prior embolizations status	0	1
Volume	5.927	0.0505

For modified AVM radiosurgery score analysis with obliteration outcome, The p-value (0.0102) indicated a significant association between modified AVM radiosurgery score groups and event outcomes. Based on Table 4, a contingency table, the group ≤1.0 has a higher observed frequency of complete obliteration event outcomes compared to other groups, suggesting that lower modified AVM radiosurgery scores are associated with higher event occurrences. The subgroup of patients classified within the modified AVM radiosurgery score range of 1.5 to 2.0 has thus far demonstrated no instances of complete obliteration following treatment. This observation may be attributed to the limited sample size, comprising only two patients, which restricts the statistical power and generalizability of the findings. Furthermore, it is important to note that the follow-up period for these patients has been relatively short, encompassing only one year post-treatment

**Table 4:** Contingency table for modified radiosurgery score association with final event. Event 0: no obliteration, event 1: complete obliteration. AVM: arteriovenous malformations.

Modified AVM radiosurgery score group	Event	
	0	1
≤1.0	1	8
1.1–1.5	0	1
1.6–2.0	2	0
>2.0	3	0

The chi-square test results for the association of prescription dose (EQD2) with obliteration status indicated a statistically significant association between EQD2 groups (<100 and 100-140) and event outcomes (0 and 1) (chi-square = 5.128, p = 0.024, df = 1). Specifically, within the "<100" EQD2 group, there were 4 individuals with no obliteration, while within the "100-140" EQD2 group, there were 9 individuals with obliteration and 2 with no obliteration. This suggests that higher EQD2 levels (100-140) are associated with a higher likelihood of event occurrence than EQD2 levels below 100.

The test for association with prior embolization showed no significant association with final obliteration.

Treatment complications

Among patients with AVMs treated with SRS/hFSRT in our study, no instances of mortality were recorded. Only one patient who had a lesion in the sensory cortex reported a touch sensory deficit over the left upper limb after SRS. Other than this no radiation-induced new neurological complications, such as hemiparesis or sensorimotor deficit were recorded or reported by the patient. Other transient complications included temporary alopecia from radiotherapy. Three patients (2%; 3/15) experienced hemorrhage following treatment. All three patients had bleeding during the latency period, of these two were large AVMs which are still patent and one was a small AVM which eventually got obliterated completely. All three patients were managed conservatively and did not require any surgical intervention. Sixty-six percent of the treated patients had new onset T2 changes (radiation-induced imaging changes, i.e., RIIC) around the nidus volume.

## Discussion

SRS has demonstrated increasing indications for its use since its introduction. This study showcases the versatility, effectiveness, and low complication rate of LINAC-based SRS for treating cerebral AVMs.

Nidus obliteration rate

The crude obliteration rate of primary AVM SRS/hFSRT treatment from our study was 60% (9/15) on MRI and/or angiogram, and 46% (7/15) on angiogram alone, which is comparable with rates in the literature for LINAC-delivered primary SRS (50-81.9%) [11]. Some studies with higher rates of obliteration (77-81.9%) excluded the outcomes of patients with under two years or incomplete follow-up [11-12]. Also, few published literature with better obliteration rates had a significant number of AVM with very small volume (<3 cc) which could have contributed to the higher obliteration rate [11,13,14], but in our study, the median volume was 3.76 cc, with 60%(9/15) cases having volume >3 cc.

The SRS-induced actuarial obliteration rates as determined by angiogram or MRI in our study at two-year, three-year, and five-year actuarial obliteration rates were 65.71%, 73.57%, and 77.14% respectively. These rates are comparable to those reported in other studies (Table 5), which documented actuarial obliteration rates from 40% to 58% at three years and 77% to 90% at five years [13,15,16].

**Table 5:** Summary of published studies; LINAC SRS of AVM. LINAC: linear accelerator, AVM: arteriovenous malformations, SRS: stereotactic radiosurgery, PF score: Pollock-Flickinger score, SM grade: Spetzler-Martin grade.

Study	System	Number of patients	Median AVM volume	Median radiosurgery dose	Obliteration rate (MRI/ANGIO)	Association with obliteration	Median follow-up in months
Mark et al., 2022 [11]	LINAC	85	4.85 cc	18 Gy	61%	Volume/dose/PF score	57
Wang et al., 2014 [12]	LINAC	116	4.67 cc	nil	81.90%	Volume/SM grade/dose	98.5
Daou et al., 2020 [13]	LINAC	112	7.4 cc	16 Gy	80.30%	Age/volume/dose/smaller isodose surface volume	67.8
Yahya et al., 2017 [14]	LINAC	47	1.97 cc	19.8 Gy	74.50%	No variable association	52
Blamek et al., 2011 [16]	LINAC	62	6.64 cc	16 Gy	35.50%	SM grade/AVM score/dose/volume	26.8
Gawis et al., 2022 [17]	LINAC	71	10.6 cc	20 Gy	66%	Dose/volume	26
Boström et al., 2016 [18]	LINAC	121	2.79 cc	19.5 Gy	71.10%	SM grade/hemorrhage/volume	43.4
Thenier-Villa et al., 2017 [19]	LINAC	195	6.9 cc	16.6 Gy	81.03%	Nil	121.91
Current	LINAC	15	3.76 cc	20 Gy	64%	Dose/volume/AVM SRS score	15

A range of factors have been identified as predictive of AVM obliteration following SRS, with marginal radiation dose being the most common factor, primarily determined by AVM volume and location [9,14,15]. In our study chi-square test revealed a statistically significant association between prescription dose, pre-procedure modified AVM radiosurgery score, and AVM nidus volume with final obliteration status. As observed in all studies the pretreatment AVM nidus volume influences the prescriptions dose, such that larger the AVM they are likely to be treated by a lower dose, and for very large volumes they are most likely to be treated by hFSRT. Similar to other published literature our study also showed that modified AVM radiosurgery score predicted accurately the percentage of patients achieving AVM obliteration [8,20].

Special cases: large AVM

AVM volume is a significant factor that determines its probability of obliteration after SRS. In our study, three patients had large AVMs, defined as having a volume larger than 10 cc. None of these patients achieved complete obliteration, and their AVMs remained partially obliterated and patent at the completion of the study.

In the published literature, researchers have recognized the challenges associated with treating large AVM, like poor obliteration rate and need for retreatment, and have suggested various approaches to treating such lesions for better outcomes. The possible options suggested are hFSRT, volume-staged radiosurgery, dose-staged radiosurgery, and endovascular embolization followed by SRS. Between the volume staged (VS-SRS) and hypofractionated radiotherapy (hFSRT), VS-SRS was found to have a higher rate of complete obliteration (40.3% vs 32.7%) but was also associated with higher post-SRS hemorrhage rates (19.5% vs 10.6%) in the latency period and higher mortality rate (7.4% vs 4.6%) in comparison to hFSRT [21,22].

In our center, we decided to treat three AVMs with volumes 28.7 cc, 22.318 cc, and 26.78 cc using hFSRT, because in the published literature most of the LINAC series used dose staged approach [23-25] and the prescribed dose was 28 Gy in four fractions. All three AVMs showed a partial obliteration with a significant reduction in their volume but none of the lesions had complete obliteration and are now deemed patent at the end of the study. The inadequate obliteration in these patients can be attributed to the lower biologically equivalent dose used for fractionated treatment compared to single-fraction treatment, as well as the influence of nidus volume.

Inferring from these outcomes the future treatment of such large AVM can be improved by delivering a higher fractionated dose, or initially treating them with Onyx embolization (Medtronic, Minneapolis, MN) and giving SRS to the post-embolization patent volume.

Limitations of the study

This study has several limitations, the sample size of the study is very low in comparison to other published literature which limits the statistical power. Also, this is a single-institution retrospective study, which could introduce potential biases. The short follow-up period in some patients is inadequate to judge the obliteration status.

## Conclusions

This study examined the outcomes of LINAC-based SRS/hFSRT for patients with brain arteriovenous malformations (AVMs), and demonstrated that the obliteration rates following primary treatment with linear accelerator stereotactic radiosurgery (LINAC SRS) were comparable to those observed in other studies. The prescription dose, modified radiosurgery score and AVM volume were found to be the primary predictors of obliteration. However, when dealing with larger AVMs, additional caution is warranted as they tend to have a lower obliteration rate. Thus an advanced LINAC which is much more available than other dedicated platforms, can be used to offer stereotactic radiosurgery treatment for cerebral AVM.
